# Tracking the follow-up of work in progress papers

**DOI:** 10.1007/s11192-017-2631-4

**Published:** 2017-12-23

**Authors:** Omar Mubin, Mudassar Arsalan, Abdullah Al Mahmud

**Affiliations:** 1 Western Sydney University, Sydney, Australia; 2 RW Corkery Pty Limited, Brooklyn, NSW Australia; 3 Swinburne University of Technology, Melbourne, Australia

**Keywords:** SIGCHI, Work in progress, Late breaking reports, Self-citations, Extending papers, CHI, HRI

## Abstract

Academic conferences offer numerous submission tracks to support the inclusion of a variety of researchers and topics. Work in progress papers are one such submission type where authors present preliminary results in a poster session. They have recently gained popularity in the area of Human Computer Interaction (HCI) as a relatively easier pathway to attending the conference due to their higher acceptance rate as compared to the main tracks. However, it is not clear if these work in progress papers are further extended or transitioned into more complete and thorough full papers or are simply one-off pieces of research. In order to answer this we explore self-citation patterns of four work in progress editions in two popular HCI conferences (CHI2010, CHI2011, HRI2010 and HRI2011). Our results show that almost 50% of the work in progress papers do not have any self-citations and approximately only half of the self-citations can be considered as true extensions of the original work in progress paper. Specific conferences dominate as the preferred venue where extensions of these work in progress papers are published. Furthermore, the rate of self-citations peaks in the immediate year after publication and gradually tails off. By tracing author publication records, we also delve into possible reasons of work in progress papers not being cited in follow up publications. In conclusion, we speculate on the main trends observed and what they may mean looking ahead for the work in progress track of premier HCI conferences.

## Introduction

Conferences, symposiums and workshops are one of the primary means of disseminating research and presenting state of the art results (Lisée et al. 2008). A typical conference is structured as a multi-day event with parallel tracks of presentations. Most full paper presentations are oral and other modes of presentation such as short papers are also witnessed depending on the content of the conference. These include shorter oral talks, demos, video presentations, poster presentations and more. Not only do conferences allow for discussion of latest findings but also provide networking opportunities for attendees. In addition, discussions held in such a platform allow researchers to explore collaborations and avenues of extending their presented work. Therefore attending conferences is an integral component of the professional duties of researchers and academics alike.

With budget deficits and financial cuts affecting the academic and research profession, researchers in most instances need to fully justify their rationale for attending a conference. Most organizations or travel grant applications require active participation, such as some form of oral presentation as the base prerequisite to support conference attendance. The size of the conference and its expected attendees is likely dependent on the number of main track or full paper presentations that take place which is related to the overall acceptance rate of the conference. The lower the acceptance rate of the conference the more difficult it may be for a larger number of researchers to justify their attendance. Most decent quality conferences only accept approximately one quarter of their submissions (Derntl 2014). Authors may also abandon thoughts of submitting manuscripts to full paper tracks at the last minute due to an inclination that their piece of research is not complete and will hence most likely get rejected (Scherer et al. 2015). However, over time, we have witnessed secondary tracks accepting paper or poster submissions of shorter length and consequently lesser content—such as work in progress submissions. These alternate tracks more often than not promote and allow presentation of snippets of research as work in progress with higher rates of acceptance through a less rigorous peer review process. This presents a win win situation for conference organizers and researchers alike (Bartneck 2010); providing the former with additional attendees and the latter with an opportunity to attain feedback on initial results and the rationale to justify their conference attendance through active participation.

Our purpose is to most certainly not doubt author publishing intentions and habits, in fact we believe that having various tracks at a conference allows researchers to be part of the community, network and both give and receive feedback. Work in progress tracks, short paper tracks, poster tracks and the like provide researchers with an opportunity to not only actively participate in the conference but more importantly present preliminary (innovative) ideas. The elementary purpose of attaining feedback on a conference presentation is the value it provides to advance the work and develop it further through additional research or collaborations. One would expect that work in progress papers would ultimately translate and transition into more established and elaborate research papers (such as conference or journal articles) after being presented at the conference.

Across most fields of research, journal articles are generally preferred in comparison to conference articles as a matter of convention. The conference versus journal debate is a longstanding one and one key concern raised against conference articles is that the research presented is not significantly polished through an iterative peer review process (Bowyer 2012). Therefore by logical extension most conference articles should be extended to journal articles, although it seems that this does not happen as often as one would expect in some disciplines (Vardi 2009). The field of scientometrics and infometrics comprises of works from other researchers aiming to analyze the follow up and nature of extension of research articles in conferences. However, most prior work has focused on the extension of full papers in academic venues [such as a simple conference to journal extension (Eckmann et al. 2011)] and discussion surrounding specifically work in progress papers is not explored. For example, previous research (Montesi and Owen 2008) has investigated the tendencies, habits and preferences of authors when extending conference articles through qualitative surveys. To the best of our knowledge there is only one quantitative study (Wainer and Valle 2013) that aims to explore the extension of Computer Science academic articles from ACM conferences and journals. We contribute towards scientomteric literature by leveraging on study of Wainer and Valle (2013) through some key advancements. Firstly, our investigation is focused on work in progress papers and not main track full papers. Secondly, although, an analysis of the type of self-citations is indicated in Wainer and Valle (2013) however in the same study only speculations are presented as to why some papers do not generate self-citations. We aimed to address both aspects in our investigation. Thirdly, in addition we also aimed to determine if the research extension trends observed were particular to our dataset or extended to other academic venues in different areas of research. In conclusion, we closely base our methodology on the study of Wainer and Valle (2013) allowing us to replicate their quantitative analysis of self-citations of conference articles on a set of work in progress articles.

As stated prior, we are gradually observing the emergence of a number of work in progress submission tracks at conferences. However, are they really an avenue of attaining feedback and extending the research or merely a solitary outcome from a research endeavour? That is, are there follow ups of such work in progress papers or are these papers written in isolation; never to be extended. Unless a work in progress paper receives negative feedback it would be difficult to assume that the embryonic research is accomplished—a possible hypothesis that could be used to explain the non-continuation of full papers (Wainer and Valle 2013). After all, most papers are expected to have a future work section (Hu and Wan 2015) but in the case of these work in progress papers does this future work really ever transpire? In this paper, we present our findings from an empirical study where we delved into the work in progress papers from four separate conference editions in the area of Human Computer Interaction to determine their follow up by recording their self-citations and type of follow up extensions. For a chosen set of work in progress papers we set out to determine the likelihood of the research being extended by measuring self-citations. Furthermore, we aimed to extract an extension pattern in work in progress papers through their rate and type of self-citations. We also aimed to check whether this rate was comparable to work in progress venues from other domains and also to the rate of extension of conference articles. We also endeavoured to deduce possible reasons for why work in progress articles are not self cited in subsequent research. In the remaining part of this paper, we summarize our data collection strategy across the work in progress venues, our main findings and their implications.

## Method

Two of the three authors have their primary research interest in the field of Human Computer Interaction (HCI) and hence we focused on collecting data from work in progress papers in 2 major HCI conferences. The Special Interest Group in HCI (SIGCHI) (SIGCHI 2017) is the premier association and body that organizes conferences in the field of HCI. The Human Factors in Computing Systems (CHI) conference is the most renowned conference from SIGCHI held on an annual basis for more than 30 years (Bartneck and Hu 2009; Mubin et al. 2017), presenting research on various aspects related to human interaction with technology. Several thousand submissions are received in the full paper main track of which only around 20% are accepted through a stringent and refereed peer review process. However, the CHI conference has since 1995 been organizing a work in progress track (albeit in different names) with the primary purpose of allowing fresh and not fully mature research a chance to be nurtured to completion (Mentis 2016). Usually, CHI work in progress papers are reviewed in a juried manner. We shortlisted the work in progress track of the *CHI 2011* (CHI Work in Progress 2011) and *CHI 2010* (CHI Work in Progress 2010) editions as the primary source of our data. The acceptance rate of the work in progress track in these editions was around 40%, with nearly 500 submissions per edition. Picking two editions would allow comparisons and we did not select very recent editions simply to account for the time it would take for work in progress papers to be extended to other richer forms of articles. Both editions in their call for papers solicited work in progress papers of 6 pages in length in the SIGCHI extended abstract template, which is essentially an abridged format. Presentation was to be made as poster form allowing for interactive discussions between researchers and attendees. Both call for papers explicitly referred to the motivation and rationale of the track as a means to attain feedback on novel work with the ultimate goal of advancing the mentioned research. We also collected data from work in progress submissions from the Human Robot Interaction (HRI) 2010 and 2011 conference (HRI 2011 Call for Participation 2011). The HRI conference is a popular SIGCHI conference having a full paper acceptance rate of also around 20%. The work in progress track of the HRI conference is termed as the late breaking reports but in essence the goals are identical to the CHI work in progress track. The HRI late breaking reports track solicits submissions in a 2 page double column ACM format.

### Data collection process

All four chosen venues (CHI2010, CHI2011, HRI2010 and HRI2011) had their work in progress papers archived in the digital library of ACM. For each work in progress paper we recorded their total citation count (*including self-citations*) and self-citation count as evidenced in Google Scholar, purely because of the citation coverage provided by Google Scholar (Meho and Yang 2007). A self-citation was treated as such if there was at least one author from the original publication listed in the follow up publication (Aksnes 2003). Using the proceedings from ACM digital library, a work in progress article was searched by title in Google Scholar and the “Cited By N” link was clicked to extract the citations. The self-citations were then extracted and counted separately. The entire data collection process was manually done. All citation data was recorded over a week in the month of May 2017. The year of the self-citation was also recorded. Each self-citation was then coded into type of publication by analyzing its bibliographic information:

1. Book
2. Book Section (or a book chapter)
3. Conference Proceedings
4. Generic
5. Journal Article
6. Report
7. Thesis

An article was placed into the “Generic” category if we could not place it in any of the other categories due to incomplete bibliographic information. Initially a process of cross checking and verification was completed. Two authors categorized papers for CHI2011 to ensure there were no reliability issues when interpreting bibliographic information; upon which a single author completed the data acquisition phase. The crux of our analysis concentrated on determining the following aspects:

1. Self-citation trends, including yearly spreads of self-citations, average number of citations and self-citations and popular venues where work in progress papers get self cited
2. The type of self-citations, that is whether a self-citation could be classified as a true extension or simply a referral in the literature review section of the follow up paper
3. Possible reasons as to why some work in progress papers do not receive any self-citations
4. Comparison with a set of work in progress papers from another area of Computer Science research to ground our findings with respect to the two HCI conferences of CHI and HRI

## Results

A number of descriptive analysis techniques were conducted to interpret our data. A table (see Table 1) is presented that summarizes the sample size and average number of total citations (including self-citations) and self-citations per paper across the four venues. Total number of self-citations for the four editions of CHI2010, CHI2011, HRI2010 and HRI2011 were 293, 244, 91 and 138 respectively. As a next step we explored the relationship between the total citations and self-citations a paper would receive. We subtracted the self-citation count from the total citation count at this stage. Thereafter, we computed means for total citations when there were no self-citations against when there was at least one self-citation (see Table 2).

### Self-citation trends

A frequency table (see Table 3) was charted that depicted the spread of self-citations for the three venues. The results showed that 42.6% of CHI2010, 44.8% of CHI2011, 48.5% of HRI2010 and 44.8% of HRI2011 submissions did not have any self-citations (a total of 78, 90, 32 and 43 papers respectively). The maximum self-citations for the CHI2010, CHI2011, HRI2010 and HRI2011 work in progress submissions were 15, 13, 6 and 10 respectively. HRI2011 had 10 work in progress papers with no citations other than self-citations (18.9% of papers with self-citations), followed by CHI2011 with 7 (6.3% of papers with self-citations) and CHI2010 with 2 (1.9% of papers with self-citations); HRI2010 had no such paper.

**Fig. 1:**
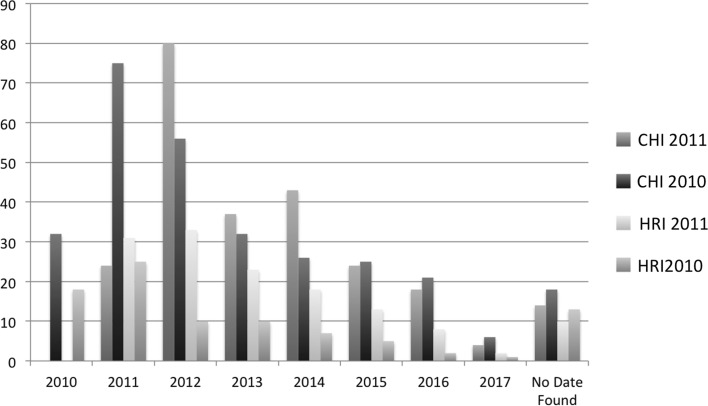
Graph of yearly trend of self-citations

**Fig. 2:**
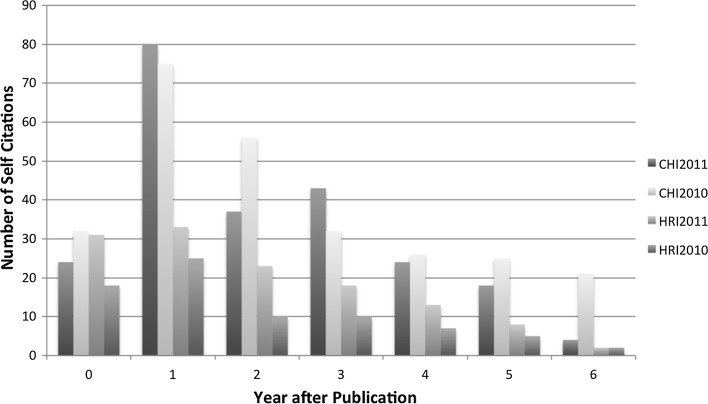
Graph of yearly trend of self-citations based on period after publication

**Fig. 3:**
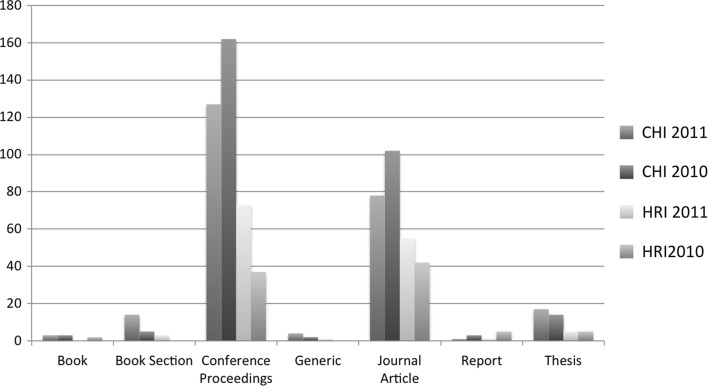
Graph of type of self-citation

**Fig. 4:**
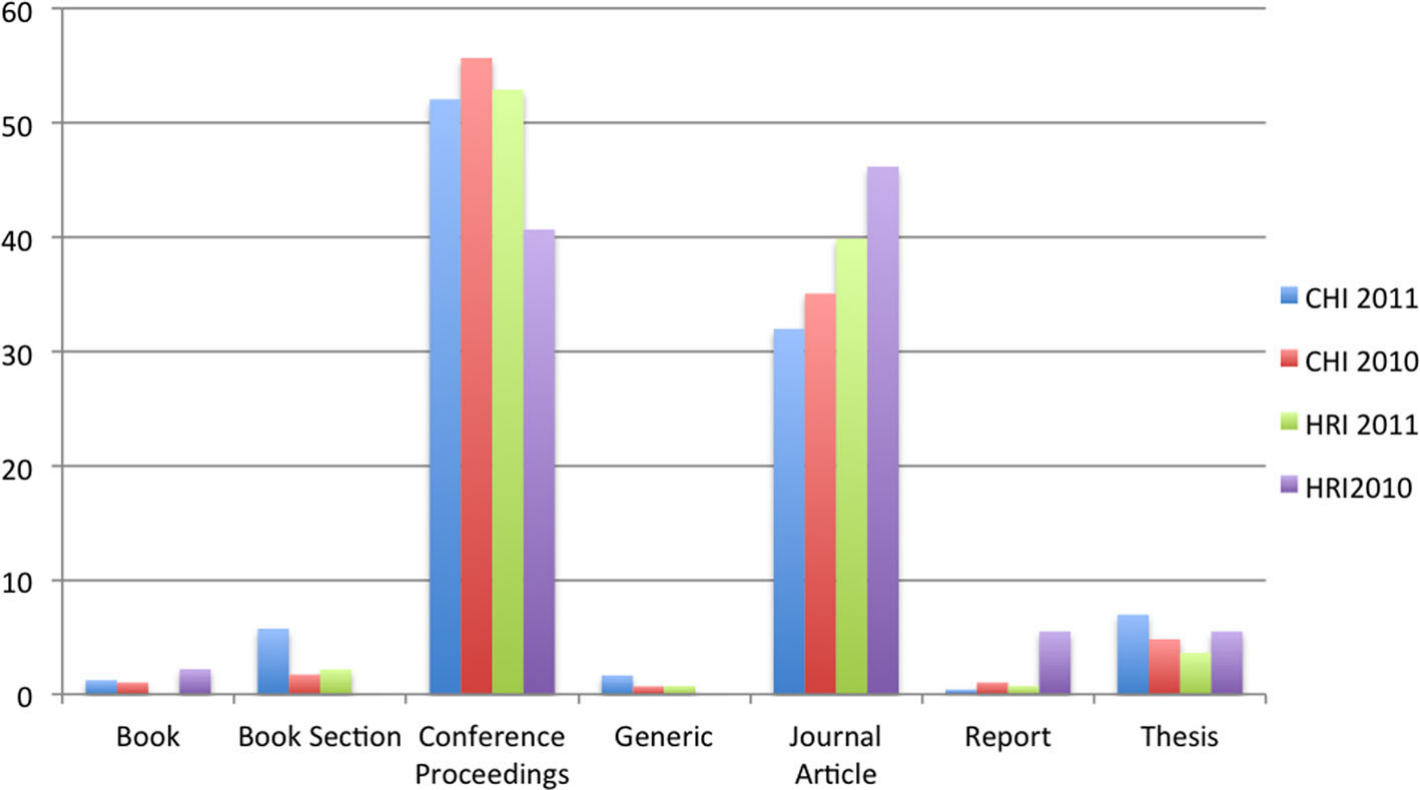
Graph of type of self-citation relative (in %) to total number of self-citations

We also plotted the yearly spread of self-citations (see Fig. 1), which showed us that in general self-citations of work in progress submission for our four editions peaked a year after publication and gradually petered out. Approximately one third of the total self-citations were attributed in the next year after publication for the four editions (see Fig. 2). An additional graph was plotted for the type of self-citations (see Fig. 3), which indicated that conference articles were the most popular type of paper where work in progress papers were cited. Yet another graph (see Fig. 4) indicated in relative terms that the proportion of conference articles and journal articles which self cite work in progress articles was very similar across the four editions.

We further explored the common venues where the work in progress submissions were subsequently cited. The top 4 most frequent venues across the four editions are presented in a table (see Table 4). Since CHI work in progress papers are located in an adjunct proceedings (separately located within ACM Digital Library and hence distinctly named as extended abstracts) they were easier to identify amongst the self-citations. At least for CHI2010 and CHI2011, it was fairly obvious that work in progress papers were being cited in subsequent CHI publications be that work in progress or full papers (both on average approximately 6%). Most work in progress papers were cited in papers from venues that were SIGCHI conferences; and journals were few and far in between (only one journal appeared as a popular follow up choice—The International Journal of Social Robotics).

### Qualitative Coding of self-citations

We acknowledge that the true reflection of whether a paper is an extension of another paper is more complicated than simply checking if one cites the other. Ideally, some form of qualitative determination would need to be carried out. In our current study, we utilized the classification coding scheme presented in Wainer and Valle (2013) as a means to dig deeper into the types of self-citations in parts of our sample. Wainer and Valle present three different types of follow ups, which are measured by analyzing the content of the citing article against the original article. These are (1) State of the Art, (2) Extension and (3) Republication. We would now like to present an example of the variation in how self-citations can present themselves. We consider two self-citations of a work in progress paper from CHI2010 (Lucero et al. 2010). In the first paper (Lucero et al. 2012) the technical platform presented in Lucero et al. (2010) is used as a testing medium to conduct further empirical research (*Extension*). Whereas in the second paper (Lucero et al. 2011) the technical platform is only mentioned in the introduction section as a means to motivate the newer form of research and state the background or related work (*State of the Art*). *Republication* as the third category is simply when the content is duplicated with minor editorial changes or change in format. We selected HRI2011 and CHI2011 as the cases of further investigation and randomly sampled approximately 25% of work in progress papers from each of the two editions, giving us a total pool of papers which was in number comparable to the sample of Wainer and Valle (2013). Self-citations of 49 CHI2011 papers and 25 HRI2011 papers were scanned in detail by the authors and disagreements were resolved by discussion. The coding process concentrated on locating the citing point within the follow up paper and then the overall influence of the cited paper as per the context was determined. A similar set of results were obtained across both conferences. There were 51 self-citations in our pool of 25 HRI2011 work in progress papers out of which 29 were deemed to be of state of the art (56.9%) and 22 as extension (43.1%). There were 80 self-citations in our pool of 49 CHI2011 work in progress papers out of which 41 were deemed to be of state of the art (51.2%) and 38 as extension (47.5%) and 1 (1.2%) as a republication.

The nature of self-citations (state of the art, extension and republication) was qualitatively determined by manual annotations as described prior. However, in an attempt to automate this process and in the endeavour of determining the textual similarity between work in progress articles and their follow up articles we carried out a pilot investigation. Using an online text similarity engine (Compare Two Documents 2017), we computed the level of similarity across work in progress articles and articles that had self-cited them (for both state of the art and extension type of self-citations). A random sample of 32 self-citations (approximately 10% of available self-citations) across HRI11 and CHI11 were considered (18 of which were categorized as Extensions and 14 as state of the art). For those self-citations that we categorized as Extensions, the range of textual similarity between the work in progress articles and their corresponding self-citations was 0.2–65.9% with a Mean of 11.7% and a Median of 4.85%. For those self-citations that we categorized as state of the art, the range of textual similarity between the work in progress articles and their corresponding self-citations was 0.2–8.6% with a Mean of 3.35% and a Median of 2.45%. An independent samples T-Test revealed that there was no significant difference between the two sets (extension and state of the art) of similarity percentages [$t(32)=1.63, p=0.11$]. Therefore, it was gradually transpiring that the true determination of the nature of the self-citation is best deduced through a contextual, semantic and qualitative examination and not through a more objective textual similarity metric.

### Analyzing papers with zero self-citations

A number of papers in our sample were attributed to having not received a single self-citation. This seemingly worrying aspect was only speculated upon by Wainer and Valle (2013), however we aimed to delve into this issue a bit further. We carried out yet another pilot investigation on a 25% random sample of CHI2011 and HRI2011 work in progress papers having 0 self-citations in order to investigate if there were any follow ups that did not cite the original paper (and if so—why). To locate any follow up research papers of the work in progress paper we checked the ACM profile page and the Google Scholar page (where available) of each author listed in the work in progress publication. We scanned their publications and attempted to find papers on similar topics through either keywords or the paper title itself. If an interesting match was found the PDF of that paper was opened to explore the content further. From our sample of 50 CHI2011 papers that had no self-citations, we observed that 10 work in progress papers had related papers which could be classified as possible extensions written by one or more of the authors of the work in progress article. Obviously the work in progress article was not cited in the follow up article. 4 of these 10 papers were published in the year 2011 itself and 5 of them were adapted and republished in conferences. There was an interesting case of a 2011 work in progress article being an extension of a 2010 work in progress article also from the CHI conference. 9 work in progress papers from the sample of 50 were the only paper on that particular topic in the ACM digital library. 4 papers that were deemed to have no extension were first authored by students; perhaps more CHI2011 work in progress papers would have been first authored by students but typically (postgrad or undergrad) students would not have an active Google Scholar or ACM author profile nor would have an online academic presence that could be easily searched.

From our deeper analysis on the sample of 25 HRI2011 papers having 0 self-citations, it was revealed that 11 of them had similarity to other papers to such an extent that the new papers could be considered as a follow up or extension. 5 of these follow ups were in the instance when the work in progress paper was emerging from a larger research program or project and 2 of these follow ups were published as work in progress articles themselves (CHI 2012 and HRI 2012). Similar to the anomaly in the CHI2011 data, there were two instances when the work in progress paper in HRI2011 was actually a follow up of a conference article from prior; in this case 2010. From the 14 papers where we could not establish an extension through our search, 5 were first authored by students (where at least one first author was currently working in an industrial capacity). In the analysis of HRI2011 work in progress papers with 0 self-citations, there were at least 3 papers where we could not trace any precedent or followup, with little to no online presence of the authors; perhaps such cases were one off projects.

### Comparison with the ICWS conference

We introduced an additional step in our analysis by collecting self-citation trends of a conference from a different research area other than HCI. Although HCI as a sub-domain emerges from Computer Science it is a rapidly evolving topic calling on discipline knowledge from a number of other (at times non-technical) areas of research (Grudin 2008). Therefore we were led to believe that the particular citation patterns depicted in HCI work in progress venues might not be mirrored in other more traditional Computer Science research areas. In addition, typically work in progress tracks are avenues to present incremental updates to systems, applications and algorithms so we focused on sourcing a conference that had such a track from any sub-discipline within Computer Science but not necessarily Human Computer Interaction. We selected the International Conference on Web Services (ICWS) (edition 2011—ICWS 2011 Work-in-Progress Track 2011) as its goals of the work in progress track were very similar to that of CHI and HRI and is also a very highly ranked conference (CORE Ranking A and a full paper acceptance rate of $<20$%). A similar data collection process was carried out as described earlier for the two Human Computer Interaction conferences, although data was collected in September 2017. From a total of 30 work in progress papers presented in the 2011 edition of the ICWS conference, 17 papers had no self-citations (56.7%). Almost half of the self-citations were found in conference articles (47%) and one third in journal papers. Similar to our previously found trends across HRI and CHI, self-citations peaked in the immediate year following publication; ICWS had 14 self-citations in the year 2012 (38.8%). The average total citations for ICWS was 7.63 (standard deviation of 8.14) and the average self-citation was 1.2 (standard deviation of 1.83).

**Table 1:** Average citation and self-citation data per paper with standard deviations in brackets

Work in progress track	Number of papers	Average citation	Average self-citation
CHI2010	183	12.14 (13.6)	1.60 (2.37)
CHI2011	201	12.34 (29.1)	1.21 (1.87)
HRI2010	66	8.06 (8.4)	1.38 (1.8)
HRI2011	96	7.10 (10.32)	1.44 (2.08)

**Table 2:** Mean total citations across self-citations with standard deviations in brackets

Work in progress track	Mean total citations when no self-citations	Mean total citations (excluding self-citations) when at least one self citation
CHI2010	7.44 (10.26)	12.84 (13.85)
CHI2011	8.37 (14.1)	13.37 (36.6)
HRI2010	5.44 (9.5)	7.85 (6.19)
HRI2011	3.91 (7.61)	7.09 (10.6)

**Table 3:** Self-citation frequency spread

# of self citations	Frequency of self-citations			
	CHI2010	CHI2011	HRI2010	HRI2011
0	78	90	32	43
1	42	58	9	22
2	25	27	12	12
3	11	9	4	8
4	9	7	3	4
5	6	1	2	1
6	5	2	4	1
7	0	3	0	2
8	2	1	0	1
9	2	2	0	1
10	0	0	0	1
11	1	0	0	0
12	1	0	0	0
13	0	1	0	0
14	0	0	0	0
15	1	0	0	0

**Table 4:** Popular venues where work in progress submissions are cited

CHI2010	CHI2011	HRI2010	HRI2011
CHI Work in Progress Track (20)	CHI Full Paper Track (20)	International Conference on Robot and Interactive Human Communication (RO-MAN) (10)	International Conference on Human Robot Interaction (13)
CHI Full Paper Track (15)	CHI Work in Progress Track (14)	International Conference on Human Robot Interaction (5)	International Conference on Robot and Interactive Human Communication (RO-MAN) (7)
Interaction Design and Children (8)	Tangible and Embedded Interaction Design (8)	International Conference on Intelligent Robots and Systems (3)	International Journal on Social Robotics (7)
Tangible and Embedded Interaction Design (5)	Conference on Computer Supported Cooperative Work (CSCW) (7)	Human Factors and Ergonomics Society Annual Meeting (3)	International Conference of Social Robotics (6)

Numbers in brackets indicate frequency; all 16 entries are conferences except for the International Journal of Social Robotics

## Discussion

In this study we have analyzed self-citation patterns of work in progress papers in four Human Computer Interaction related conferences from the special interest group SIGCHI; namely CHI2010, CHI2011, HRI2010 and HRI2011. Our results show that almost 50% of the papers do not have any self-citations and apparently no extension. This is comparable to prior work that investigated the follow up of conference articles (Wainer and Valle 2013; Eckmann et al. 2011). However the thinking behind the work in progress track in both CHI and HRI is to promote discussion and future extensions of research, so in reality we should expect a higher self-citation rate as compared to more mature conference articles. This is clearly mentioned in the call for papers for CHI2011 (CHI Work in Progress 2011): “A significant benefit of a Work-in-Progress derives from the discussion between the author and conference attendees that will be fostered by the face-to-face presentation of the work.” Almost half of the articles that we considered in our sample are not cited again by their authors in newer papers. Could this indicate that researchers are using the work in progress track as a means to justify attendance to a flagship conference; or is the work in progress track being used as a means to test the waters so to speak, gauging the community’s interest in new ideas and concepts. If it is the latter, we would expect a higher self-citation rate.

We also speculate that at times some authors do not cite their work in progress papers when extending the work since occasionally in work in progress papers authors own the copyright (as indicated by our pilot analysis of CHI2011 and HRI2011 papers with 0 self-citations). Both CHI2010 and CHI2011 are explicit in their copyright ownership regulations clearly specifying that it belongs to the authors and text from work in progress papers can be used in further publications as long as they are *significant revisions*. HRI2011 interestingly provides an opt out option where authors can choose to have their work in progress papers to be not archived in the ACM Digital Library. This caveat should not have affected our results as we only considered HRI2011 papers that were available in the ACM Digital Library. The clear reason why authors would not cite their work in progress article is difficult to determine with conformity but we did observe a number of replication trends—such as using the exact figures and data or indirect extensions (papers on roughly the same topic but with different research processes or outputs). The nature of the work in progress track both at CHI and HRI is such that it allows a publication opportunity for incremental projects (such as student projects undertaken in the course of a semester). As mentioned prior, accurately determining how many papers were driven by the work of students (undergrad or postgrad but not PhD) is difficult but this should be considered as a plausible reason for the research presented in work in progress papers to not be extended. Students would either graduate or finish their project and would not need to extend or carry it further. Other reasons include one-off attempts at getting projects published or in the other instances when the work in progress paper is part of a larger research program, where the authors do not deem necessary to cite the work in progress paper in the follow up.

Our dissection of a random sample of CHI11 and HRI11 self-citations revealed that when self-citations did eventuate around half of them could be only considered as true extension of the work in progress article. This was realized through the qualitative analysis of self-citations. It was usually fairly simple to identify whether the new research was being extended on the basis of the work in progress article as the authors were very explicit—either by using terms such as “initial results are presented in”, “in extension of”, etc. There was even one instance when in the Acknowledgement section of the follow up paper the following sentence was added: “This paper is a heavily revised and extended adaptation of a previous CHI work-in-progress publication.” We also witnessed on at least 3 separate occasions, where the follow up publication was a subsequent extended iteration in the first author’s PhD candidature (and hence a citation to the preceding work in progress article was expected). In other instances the work in progress article presented the design and/or implementation or pilot evaluation of a specific module of a larger computing system, which was then rigorously evaluated or fully developed in the follow up research. In summary, when the follow up paper was a true extension of the work in progress article the linkage or relationship between the two was established through various means and sources; such as sharing the data source or platform/tool or even at times the methodology. When the follow up paper cited the work in progress paper in the related work section or as state of the art in most instances the author did not attempt to signify that this was their previous piece of research (or did so in third person)—a clear indication that they did not want the new paper to be linked to the previously published work in progress paper. Furthermore, a mini-investigation of sorts on a self-citation sub-sample illustrated that textual similarity levels across the two types of citations were not significantly different. In summary, authors adopted various techniques and norms to link their new pieces of research with previously published work in progress articles.

We also noticed that up to 50% of the self-citations were located in conference articles. Human computer interaction is a sub-discipline of Computer Science, where conferences such as the CHI conference are of international repute. Therefore we can expect that authors should be comfortable in extending their work in conferences (Vardi 2009; Freyne et al. 2010). Most of these venues were from SIGCHI and interestingly subsequent work in progress editions were considered a viable option to submit the extended research (34 self-citations of CHI2010 and CHI2011 papers were in subsequent CHI work in progress papers). The rate of self-citations of work in progress papers was on average highest in the immediate year following publication. This is further exemplified by the average time for CHI work in progress to appear as self-citations in the CHI full paper track was 1.65 years for CHI2011 and 1.60 for CHI2010. In addition, we can only speculate if the nature of the research presented and discussed in work in progress papers is such that it is most suited to SIGCHI venues and not other journals or conferences. Both HRI and CHI have associated journals (TOCHI and JHRI-now renamed as ACM Transactions on HRI), of whose accepted papers are presented orally at the conference. We may have anticipated that such journals would have been appropriate venues to publish extended work from work in progress papers. However both journals were underrepresented, TOCHI only appeared once as a venue where a work in progress paper was self cited in CHI2010, four times in CHI2011 and JHRI only had 2 relevant self-citations. Thesis publications were the third most popular source of self-citations, which probably indicates that the work in progress track is a popular presentation outlet for Master or PhD students. For such students the possibility of multiple subsequent publications maybe low (there will only be one thesis) thereby reducing the overall rate of self-citations.

We utilized the ICWS2011 edition was a means to ground our findings obtained from analyzing the two CHI and HRI work in progress tracks. Although the two CHI editions seem to have higher impact as realized through the citation rate, the trends across the entire data set were similar; with the three (ICWS, CHI and HRI) conference tracks indicating a generally low level of follow up (particularly in journals) as one could expect with such an exploratory paper type. This mirroring lends some credibility to our overall results. Most self-citations in the CHI and HRI conferences were observed to exist in SIGCHI conferences and although such venues are reputable [with high CORE rankings (confportal 2016)], perhaps the SIGCHI association can promote and advertise additional special issue journals where submissions of extensions of work in progress papers are encouraged.

As a final discussion point we would like to relate our results with those of Wainer and Valle (2013). Although their research was based on the extension of Computer Science research articles (both conferences and journals); we attempt to summarize some of our key results by directly comparing them to those from Wainer and Valle (see Table 5). The rate of a paper having no follow ups whatsoever was similar (see row 2 of Table 5). Although the rationale behind a work in progress track (which was our focus) is different from a full paper track (which was the focus of Wainer and Valle 2013) the mirroring of these results is interesting. True extensions of papers across both studies were about half of the total self-citations (see row 4 of Table 5). Work in progress papers in our sample were cited less frequently in journals in comparison to full papers analyzed by Wainer and Valle (2013) (see row 6 of Table 5), but this may just be a tendency of researchers in the field of HCI to prefer publishing in SIGCHI associated conferences.

**Table 5:** Comparing our results with Wainer and Valle (2013)

Measure	Work in progress articles (HRI and CHI) (%)	Wainer and Valle: computer science publications (%)	
		Conference	Journal
No follow-ups: 0 self-citations	45.2	44	51
Type of self-citation			
State of the art	53.4	44.9	57.1
Extension	45.8	43.3	42.9
Venue of self-citation			
Conference	52.2	50.4	57.1
Journal	36.2	49.6	42.9

## Limitations and conclusion

We would like to acknowledge certain limitations of our presented results. Firstly, by no means is our sample size comprehensive and complete. We have only considered data from four particular editions of the CHI and HRI conferences and as such our data is susceptible to tendencies of the field. However, as stated prior our rationale of considering editions from 2010 or 2011 was to provide enough time for the papers to mature. In retrospect, our results did show that most self-citations take place in the immediate year after publication. We did attempt to generalize our results by running a similar analysis on a conference from another area of research, however we have only compared results from the two HCI venues to only a single edition of the ICWS work in progress track (2011). In the future it maybe worthwhile to consider work in progress papers from other areas of Computing research and to even investigate extension patterns of work in progress papers from other HCI venues; such as the Designing Interactive Systems conference (DIS).

Secondly, we acknowledge that some work in progress research maybe extended into longer articles implicitly without citations taking place and we have tried to dissect this possibility by coding a sub-sample of our data. Thirdly, we acknowledge that it is challenging to determine the exact reasons why a project described in a work in progress paper is not extended unless the authors themselves are contacted or surveyed. Whilst we have attempted to dissect and track the trail of a work in progress paper post publication, we have only considered a random sample of CHI2011 and HRI2011 work in progress papers—in line with the work of Wainer and Valle (2013). Consequently, our speculations with regards to why work in progress papers are not cited in follow up publications must be treated with caution. Lastly, given that our data gathering process from Google Scholar was manual in nature and although the extraction was carried with utmost care, unintentional omissions or errors might have been introduced.

In conclusion, we believe that the self-citation rate of work in progress papers appears to be low as exemplified through our data sample. A deeper analysis revealed that true extensions of work in progress articles were about half in number of the total self-citations. We also closely scrutinized a sub sample of our data set with no self-citations and found between 20 and 50% papers were extended but not cited. Reasons for not citing may simply be an issue of the author owning the copyright or double blind peer reviewing requirements. The work in progress track of conferences is designed to function as an interactive track providing quick and real time feedback to participants on initial results. As such it is an ideal platform for incremental pieces of research which may be accomplished by smaller research groups or even students. This could be one additional and plausible explanation of why many work in progress papers are not extended. As observed in our sample, most work in progress articles are cited by their own authors in conference papers only. This may simply be a tendency that HCI researchers have; which is a preference towards SIGCHI conferences as the first “go to” venue to publish their research. Specifically the HCI research community probably requires pathways, guidelines and guidance from the SIGCHI body in advancing their work in progress articles to more extensive pieces of research.
